# Three-Way Interaction Effect Model: Moderating Effect of Resource Between Business Age and Host Performance

**DOI:** 10.3389/fpsyg.2021.763633

**Published:** 2021-11-25

**Authors:** Xiaobei Liang, Li Tang, Zhen Xu, Xuanxuan Lyu

**Affiliations:** ^1^School of Economics and Management, Tongji University, Shanghai, China; ^2^School of International Studies, University of International Business and Economics, Beijing, China

**Keywords:** micro-entrepreneurship, accommodation sharing, resource-based theory, host performance, business age

## Abstract

In the field of accommodation sharing, little attention has been paid to micro-entrepreneurship of hosts. Based on the signaling theory and the resource-based theory, we proposed a three-way interaction effect model to investigate the moderating effect of resource configuration (business size and host reputation) on the relationship between business age and host performance. A statistical analysis of the secondary panel data crawled from Airbnb.com was tested through the negative binomial model. The results shown that: (1) Business age is positively related to host performance; (2) the positive impact of business age on host performance is stronger for smaller size; host reputation has no significant moderating effect on the relationship between business age and host performance; (3) the joint consideration of business age, size, and host reputation has a three-way interaction effect on host performance. The positive impact of business age on host performance is strongest for hosts with smaller size and higher host reputation. These results are helpful to understand the micro-entrepreneurship performance of hosts in the field of accommodation sharing.

## Introduction

In recent years, the sharing economy has witnessed a spurt of development on a global scale. It has significantly changed the tourism and hospitality industries (Priporas et al., 2017; Akbar and Tracogna, 2018). Since tourism and hospitality industries are labor-intensive requiring less expert knowledge (Phizacklea and Ram, 1995), they have become one of the main areas of individual entrepreneurship (Hollick and Braun, 2005; Nikraftar and Hosseini, 2016; Li et al., 2020). The emergence of P2P accommodation platforms provides individuals with “franchising” micro-entrepreneurship opportunities (Cohen and Sundararajan, 2015). Individuals are able to generate income by renting out vacant houses through P2P accommodation platforms (Martin, 2016; Teubner et al., 2017). Airbnb, the premier platform for shared accommodation (Oskam and Boswijk, 2016), has attracted 4 million people in 100,000 cities worldwide to rent out their vacant houses for micro-entrepreneurship (Airbnb, 2021).

Previous studies have focused on the economic and social impact (Oskam and Boswijk, 2016; Koh and King, 2017), marketing and consumer behavior (Lutz and Newlands, 2018; Casais et al., 2020), and pricing issues (Gibbs et al., 2018; Magno et al., 2018) on accommodation sharing. However, to the best of our knowledge, fewer studies have regarded Airbnb hosts as the micro-entrepreneurs, and thus, the research on accommodation sharing entrepreneurship has been largely neglected (Sigala, 2016). Against the backdrop, this study explored the accommodation sharing from the perspective of micro-entrepreneurship. Tourism micro-entrepreneurship is small-scale, informal tourism businesses with fewer owners/managers/worker (Ferreira et al., 2015; Ditta-Apichai et al., 2020). In this perspective, each host is all operating a micro-enterprise. As the competitive pressure is increasing with the number of hosts on the platform increases, it has become an important issue to explore the impact mechanism of the host’s performance.

Existing studies have identified the significant impact of firm age on firm strategy and performance (e.g., Steffens et al., 2009; Ismail and Jenatabadi, 2014). Nevertheless, consensus on how firm age affects firm performance has not yet reached and two major views are found in literature. The first propose is that younger firms are more risk aware (Shane and Venkataraman, 2000), flexible (Resnick et al., 2006), and innovatively and thus could effectively utilize their own resources to achieve better performance (Withers et al., 2011; Kilenthong et al., 2016). On the other hand, the second view holds that the managers of young firms are lack of experience and knowledge (Slevin and Covin, 1997; Thornhill and Amit, 2003), which then leads to misuse and waste of resources. As a firm grows maturer, its experience and knowledge become progressively abundant, and thus, its performance improved (Harvie et al., 2010). There are two main reasons for this controversy: First, the discussion of the relationship between firm age and performance lacks a specific industry analysis; second, the impact of resources on this relationship is not clearly resolved. Therefore, this study attempts to answer the following two questions:

On the sharing accommodation platform, how does business age affect host performance?

How does the host’s resource configuration (business size and host reputation) affect the relationship between business age and performance?

To answer the aforementioned question, a three-way interaction model was proposed based on the resource-based theory. Specifically, this study investigated the moderating influence of host’s business size and reputation on the relationship between business age and host performance by using Airbnb’s secondary panel data. Random effect negative binomial models were adopted to test hypotheses. This paper examined the following issues: (1) the direct effect of business age on host performance; (2) the moderating effect of size and host reputation on the above direct effect; and (3) the three-way interaction effect of business age, size, and host reputation on host performance. The findings not only enrich the resource-based theory and provide implications for future study on hosts’ entrepreneurial behavior, but also help hosts to optimize performance or make business expansion decisions.

## Theory and Model

### Signaling Theory

The signaling theory proposed by Spence (1978) provides a basic framework to account for how buyers and sellers try to solve the problem of information asymmetry in the online shopping context. Sellers have an incentive to signal the quality of their goods and services to customers. When customers are unfamiliar with service providers, they can use information clues, such as the service provider’s past experience as the reflection of service quality (Wells et al., 2011). External signals (Richardson et al., 1994) are usually not evaluated as the inherent clues of the product, such as sellers’ reputation (Chu and Chu, 1994) and commitment (Boulding and Kirmani, 1993), and are generally considered to be better received than internal signals (i.e., product clues), especially when external signals are more accessible or easier to understand (Parasuraman et al., 1988). In general, signal sources with high reliability are more effective in changing users’ attitudes or behaviors than that with low reliability. When users believe that signal providers have rich relevant knowledge, experience, or resources, users are more likely to adopt their signal for decision-making (Wu and Wang, 2011). Similarly in the field of sharing accommodation, if the hosts show more resources and professionalism, it will usually be easier for consumers to make decisions (Xie et al., 2021).

### Resource-Based Theory

The resource-based theory stresses that internal resources of a firm are the primary determinants of a firm’s superior competitive advantage (Wernerfelt, 1984; Barney, 1991). Resources refer to the tangible and intangible resources used by the firm to conceive and implement its strategy (Barney, 2001; Wiklund et al., 2010). The former include financial or physical value (Grant, 1991), while the latter refers to non-physical resources, such as skills, reputation that brings firms sustainable competitive advantages (Hall, 1992; Amit and Schoemaker, 1993). Firm resources are essential for the creating, implementing, and obtaining entrepreneurial behavior rewards (Covin and Slevin, 1991). Small- and medium-sized enterprises (SMEs) may be particularly restricted in terms of tangible and intangible resources (Thornhill and Amit, 2003; Anderson and Eshima, 2013).

Both tangible and intangible resources are crucial for a company to gain a competitive advantage (Williamson, 1975; Barney, 1991; Tayles et al., 2007)). Previous studies revealed that tangible resources (e.g., financial and fixed asset) are significantly positively related to entrepreneurial orientation or firm growth. For instance, firm size, as an indicator of the stock of tangible resources (Wiklund and Shepherd, 2003; Audia and Greve, 2006), has been proved to be significantly related to firm performance, particularly entrepreneurial performance (Rajan and Zingales, 1995; Serrasqueiro and Nunes, 2008). However, Kamasak (2017) found that intangible resources make a larger contribution to firm performance than tangible resources. This is especially true for SMEs, that is, SMEs with higher levels of intangible resources have greater flexibility, which may promote firm growth (Rogers, 2004). Firm reputation, for example, is considered as a critical intangible resources in a firm’s strategic arsenal (Barney, 1991) and has been proved to be positively correlated to firm performance (Greenwood et al., 2005; Hall and Lee, 2014).

### Business Age and Host Performance

Though firm age, as mentioned above, can significantly impact the firm’s strategy and performance (Stinchcombe, 1965; Kristiansen et al., 2003), the empirical conclusions are controversial on the relationship between firm age and performance. Following Birch’s (1979) pioneering research, the mainstream view is that younger firms grow faster than older ones (Geroski and Gugler, 2004; Davidson, 2010). For instance, the study by Coad and Rao's (2010) study found that the expected growth rate of sales, profits, and productivity of older firms was low, and they have not converted employment growth into sales, profits, and productivity growth. However, research by Coad and Rao (2010) and Capasso et al. (2015) also found evidence that firm performance increases with age. This result is grounded on the consensus that older firms have more specific resources than younger firms (Hannan and Freeman, 1984; Ranger-Moore, 1997). Specifically, in the start-up stage, human capital like the knowledge and operating ability of the manager or founder is critical for the survival of the business (Van Praag, 2003). In the following stages, manager’s capacity in older firms can be increased through previous start-up or management experiences. In this sense, compared with the younger firms which lack knowledge or resources to execute their strategy (Venkataraman et al., 1990; Lussier, 1995), older firms are likely to perform better with the growth in manager’s experience and knowledge (Harvie et al., 2010).

Knowledge and intelligence accumulated through interaction with consumers is one of the critical factors for firms to succeed in the competition within tourism and hospitality industries (Stamboulis and Skayannis, 2003). For hosts under analysis in this study, most of them are non-accommodation professionals who seek for extra income by utilizing idle resource (Lee, 2016; Li et al., 2019). That is, they have not received professional training and lack experience and knowledge in customer management, cost control, platform recommendation algorithms, and taxation (Dillahunt and Malone, 2015; Cetin and Bilgihan, 2016; Hamari et al., 2016; Jhaver et al., 2018; Liang et al., 2020). However, through learning by doing, they can continuously improve their skills in terms of using facilities, tools and technology, cost reduction, pricing, and interaction with guests (Wang and Nicolau, 2017; Benítez-Aurioles, 2018). This also enables the host to replicate its service operations more effectively, further improving service profitability. That is, with the host’s business age increasing, the profitability of host is more likely to increase (Agiomirgianakis et al., 2012; Aissa and Goaied, 2016). In addition, time is one of the key contributors to the development of social capital (Nahapiet and Ghoshal, 1998). For the sharing economy, the social capital that hosts build over time may positively affect consumer satisfaction and trust (Huang et al., 2017; Teubner et al., 2017), so the host performance therefore will accordingly getting better and better. Based on above, we propose the following hypothesis:

*Hypothesis 1:*The host’s business age is positively associated with host performance.

### The Moderating Role of Size

Previous research believes that the influence of firm age on firm performance is different according to resources obtained (Steffens et al., 2009). Most of those studies explored the boundary conditions. For example, many scholars discuss the interactive impact of firm size and firm age on firm growth or performance (Steffens et al., 2009; Nunes et al., 2013). Specifically, larger enterprises generally have scale-based cost advantages, while SMEs with less assets show better flexibility, enabling them quickly to respond to market changes (Lam et al., 2019; Choi et al., 2021). In the early stage of entrepreneurship, insufficient funds or managers’ capabilities are some of the main reasons for the SMEs’s death (Wiklund et al., 2010). In this sense, for SMEs, larger size may increase their fund pressure and management costs, and as the firm age grows, it may hinder the improvement of managers’ experience and ability, which then affect the growth in performance.

As an emerging industry, sharing accommodation provides more opportunities for early hosts to build first-mover advantages. In the stage of initial entry, hosts entering the sharing economy platform may carry out particular renovations before renting, such as decorating and adding new facilities, which may increase funds burden. In addition, as hosts are mostly self-employed, large size means that they are required to manage the multiple accommodations at the same time, which can not only increase the management and service costs, but also affect consumers’ perception of service quality. As a result, consumer satisfaction and trust may decline, which can weaken the effect of age on performance. Thus, for small size businesses, hosts are more flexible in responding to the market and can pay more attention to service quality. With the increase of business age, hosts’ management and service capabilities grow faster, which then is more likely to promote performance growth. On the country, for large size businesses, management and service costs are higher. The management capabilities accumulated over time may not be enough to respond to market changes and satisfy consumers, which may weaken the positive impact of age on performance. Based on above, we propose the following hypothesis:

*Hypothesis 2:*The relationship between business age and host performance is moderated by size, such that the relationship is stronger for lower size.

### The Moderating Role of Host Reputation

Under resource-based theory, intangible resources are not easy to obtain and replicate in the factor market, which is the most potential source of business success (Kor and Mesko, 2013; Molloy and Barney, 2015). As one of the typical intangible resources, firm reputation involves a comprehensive external evaluation of the firm’s past performance (Dowling, 2016). Moreover, due to the inherent uncertainty associated with Internet transactions, firm reputation is considered a key asset for online sales (Bensebaa, 2004), which can inform consumers of the credibility and quality of the company, thereby simplifying the consumer’s decision-making process (Drover et al., 2018). Therefore, reputation is considered as the key driving force for consumers to respond positively to the firm and has positive effects on firm performance (Srivastava et al., 2001; Wei et al., 2017).

According to the signaling theory, the comprehensive evaluation of hosts’ reputation, such as “superhost” in the Airbnb platform, indicates that the host’s comprehensive service quality is relatively high. Previous studies have shown that online users are more willing to use and pay higher fees to service providers with high reputation scores (Yacouel and Fleischer, 2012; Ert et al., 2016). “Superhost” is considered more trustworthy and high-quality which can attract more consumers and orders (Kim et al., 2016; Viglia et al., 2016). That is, if the host’s reputation is high, as business age grows, the accumulation of word-of-mouth may make it easier for businesses to gain performance growth. Therefore, we propose the following hypothesis:

*Hypothesis 3:*The relationship between business age and host performance is moderated by host reputation, such that the relationship is stronger for higher host reputation.

### Business Age, Size, Host Reputation, and Host Performance

Grant (2002) stated that selecting strategically relevant tangible and intangible resources to generate more value is the best way to attain superior sustainable performance. This study proposes that tangible resources and intangible resources may synergize in the host entrepreneurship process, which brings performance growth. In light of the relationship between business age and host performance, business age is more suitable for hosts with a smaller size and a higher level of host’s reputation. This is because that smaller business size and higher host reputation would create synergy in business age implementation as internal resources from highly consumer identification of host’s reputation can enhance the complementarity in the influence of business age on host’s performance, thereby further enhancing the fit between smaller business size and business age.

The resource-based theory indicates that valuable, rare, and unique resources promote favorable performance results outcomes (Wernerfelt, 1984; Barney, 1991). Newbert (2007) observed that intangible resources are the critical source of competitive advantage due to their inherent inimitable nature. Combined with Hypotheses 2 and 3, this shows that the relationship between business age and performance would be the strongest among smaller size of hosts that also possess a host reputation advantage compared with industry peers. That is, hosts with smaller-sized business are likely to respond to market changes faster and pay more attention to single consumers with higher service quality than their bigger-sized peers. Meanwhile, compared with hosts of larger-sized business, hosts with smaller-sized ones may make better use of their host reputation with strategic value to form competitive advantages. Therefore, they show the strongest performance growth.

In a netshell, as business size decreases, the fit between business age and host performance increases, which can be further improved by high host reputation. Accordingly, the positive impact of business age on host performance will enhance for smaller size as host reputation increases. Therefore, we propose the following hypothesis:

*Hypothesis 4:*Business age, size, and host reputation have a three-way interaction effect on host performance, such that the association between business age and host performance will be strongest when size is smaller and host reputation is higher.

## Materials and Methods

### Data and Measures

Airbnb was selected as the data set, which is the world’s largest peer-to-peer (P2P) accommodation sharing platform (Nieto García et al., 2020). Airbnb is growing rapidly, with over 5.6 million worldwide listings in nearly 220 countries and roughly 900 million arrived guests (Airbnb, 2021). There are almost 4 million hosts on Airbnb with $9,600 average annual earnings per host (Airbnb, 2021). We extracted all Airbnb listings information in Beijing, a representative Chinese city with a long history of accommodation sharing. Every host of Airbnb has a unique profile page that contains the release date, all guests’ reviews, and their attributes. By sorting these time clues, we track the host’s start-up and growth behavior over time. Since this study focused on the influence of business age on hosts’ total booking behavior, we studied hosts in the platform during the period 2013–2018 to ensure the robustness of the results. After eliminating hosts that do not exist for the entire 5years, the final panel data including 348 hosts from May 2013 to May 2018 were identified.

Table 1 shows the definition of variables and summary statistics. The dependent variable is the total number of reviews for all properties (*Host_Num*) operated by a host. This variable indicate the popularity of the host and represent the booking behavior of the host (Liang et al., 2020). There are several reasons for choosing this to represent the host performance. First, the total number of reviews reflects the lowest booking threshold. This is because that the guests are able to post reviews on Airbnb only after completing the booking. Second, as Liang et al. (2017) states, the unique design of the accommodation sharing platform makes the review volume a crucial predictor of total bookings. Third, previous researches show that most of the reviews on Airbnb are positive with ratings higher than 4.5 (the full scale is 5), so review valence is not a necessary consideration (Fradkin et al., 2015). The three-way interaction variables are the years since a host joined the platform (*Business_age*); the number of properties (Size) operated by a host; and superhost status (*SuperHost*) represents the reputation of the host. We also have control variables of host characteristics that may affect the host performance, including the average time a host takes to confirm to customer reservations (*ConfirmTime*); whether the host has identity verifications on the platform (*IdentityVerified*); and whether the host has a detailed self-introduction (*HostDescribe*).

**Table 1:** Descriptive statistics.

Variable	Definition	Mean	SD	Min	Max
Host_Num	Number of reviews a host has received	6.92	16.65	0.00	209.00
Business_age	Number of years since a host registered with Airbnb	2.46	1.63	0.00	8.00
Size	Number of properties operated by a host	0.68	1.18	0.00	10.00
SuperHost	Dummy variable indicating whether the host is recognized by Airbnb as a superhost, with values of 1=Super Host, 0=Regular Host	0.31	0.46	0.00	1.00
ResponseTime	The average time a host takes to confirm to customer reservations, with values 1=More than a day, 2=One day, 3=Few hours, 4=Less than an hour	3.49	0.85	1.00	4.00
HostDescribe	Dummy variable indicating whether the host has a detailed self-description, with values of 1=Described, 0=Not Described	0.78	0.41	0.00	1.00
IdentityVerified	Dummy variable indicating whether the host has identity verifications on the Airbnb, with values of 1=Verified, 0=Not Verified	0.75	0.43	0.00	1.00

### Model Specification

The dependent variable is count data with non-negative integers (number of reviews a host has received); thus, this study takes into consideration Poisson regression and negative binomial regression. However, as the conditional variance is much larger than the conditional expectation and data presented are overdispersed, Poisson regression is rejected. After Hausman tests, random effect negative binomial models with year are established to examine the significant role of unobserved host characteristics (Yao et al., 2020). In order to test the three-way interaction effect of *Business_age*, *Size*, and *SuperHost* on *Host_Num*, this study analyzed three βmain equations as below:


$$\mathrm{Host}\_{\mathrm{Num}}_{\mathrm{it}}=\alpha_{0}+\beta_{1}\text{Business}\_{\mathrm{age}}_{\mathrm{it}}+\beta_{2}{\text{ResponseTime}}_{\mathrm{it}}+\beta_{3}{\text{IdentityVerified}}_{\mathrm{it}}+\beta_{4}{\text{HostDescribe}}_{\mathrm{it}}+\mu_{i}+\lambda_{t}+E_{\mathrm{it}}\#\left(1\right)$$

Host_Numit=α0+β1Business_ageit+β2logSizeit+β3SuperHostit+β4Business_ageit∗logSizeit+β5Business_ageit∗SuperHostit+β6logSizeit∗SuperHostit+β7ResponseTimeit+β8IdentityVerifiedit+β9HostDescribeit+μi+λt+Eit#2

Host_Numit=α0+β1Business_ageit+β2logSizeit+β3SuperHostit+β4Business_ageit∗logSizeit+β5Business_ageit∗SuperHostit+β6logSizeit∗SuperHostit+β7Business_ageit∗logSizeit∗SuperHostit+β8ResponseTimeit+β9IdentityVerifiedit+β10HostDescribeit+μi+λt+Eit#3

where $\mu_{i}$ and $\lambda_{t}$ denote the individual effects and time effects, $\alpha_{0}$ is the constant term, and $E_{it}$ represents the residual error term. We take a log transformation on the *Size* with skewed distribution (skewness=3.267). In equation (1), we estimate whether *Business_age* has a positive impact on *Host_Num*. In equation (2), we estimate whether *Size* and *SuperHost* have the moderating influence on the relationship between *Business_age* and *Host_Num*. In equation (3), we estimate under what level of *Size* and *SuperHost*, the relationship between *Business_age* and *Host_Num* is the strongest. Since there may be multicollinearity between the interaction terms, variables in the interaction terms have been mean-centered, and models have been calculated the variance inflation factor (Day and Wensley, 1988).

## Results

Table 2 shows the correlation coefficients among all variables. The results show that *Business_age* (*r*=0.240, *p*<0.01), *Size* (*r*=0.553, p<0.01), and *SuperHost* (*r*=0.181, p *<* 0.01) are significantly positively correlated with the dependent variable *Host_Num*. Also, the variance inflation factor (VIF) of all models are tested to estimate the multicollinearity. The results show that the average VIF value is 1.45, which is lower than 10, indicating that multicollinearity is not a serious problem in the study (Mason and Perreault Jr., 1991).

**Table 2:** Correlation coefficient matrix.

	1	2	3	4	5	6	7
Host_Num	1.000						
Business_age	0.240^***^ (0.000)	1.000					
log(Size)	0.553^***^ (0.000)	0.590^***^ (0.000)	1.000				
SuperHost	0.181^***^ (0.000)	0.028 (0.242)	0.015 (0.537)	1.000			
ResponseTime	0.122^***^ (0.000)	−0.065^***^ (0.007)	−0.022 (0.362)	0.239^***^ (0.000)	1.000		
HostDescribe	0.098^***^ (0.000)	0.088^***^ (0.000)	0.093^***^ (0.000)	0.155^***^ (0.000)	0.001 (0.981)	1.000	
IdentityVerified	0.042^*^ (0.079)	0.026 (0.273)	0.004 (0.878)	0.133^***^ (0.000)	0.019 (0.418)	0.129^***^ (0.000)	1.000

*Values of pin parentheses*.

* *p<0.1*;

*** *p<0.01*.

In Table 3, main models 1 to 3 show the coefficients of the random effects negative binomial estimations. In model 1, *Business_age* and control variables were introduced. The results suggest that *Business_age* has a significantly positive effect on *Host_Num* (*β* = 0.147, *p* <0.01). Hypothesis 1 was supported. It indicates a general understanding that with the accumulation of experience, the host has enhanced business capacities, entrepreneurial experience, and social capital, which are all conducive to improving their business performance.

**Table 3:** Regression result.

	Negative binomial regression (Main Models)			OLS regression (Robustness Models)		
	Model 1	Model 2	Model 3	Model 4	Model 5	Model 6
Constant	−4.591^***^ (0.34)	−4.848^***^ (0.27)	−4.596^***^ (0.28)	−1.086^***^ (0.17)	−0.649^***^ (0.11)	−0.619^***^ (0.11)
ResponseTime	0.283^***^ (0.07)	0.243^***^ (0.05)	0.239^***^ (0.05)	0.248^***^ (0.04)	0.165^***^ (0.03)	0.163^***^ (0.03)
HostDescribe	0.373^**^ (0.15)	0.157 (0.1)	0.161 (0.1)	0.190^**^ (0.09)	0.061 (0.05)	0.061 (0.05)
IdentityVerified	0.13 (0.12)	−0.057 (0.1)	−0.048 (0.10)	0.085 (0.09)	0.017 (0.05)	0.019 (0.05)
Business_age	0.147^***^ (0.06)	0.437^***^ (0.07)	0.346^***^ (0.07)	0.429^***^ (0.02)	0.152^***^ (0.02)	0.137^***^ (0.02)
log(Size)		3.759^***^ (0.2)	3.473^***^ (0.26)		2.836^***^ (0.15)	2.666^***^ (0.17)
SuperHost		0.648^***^ (0.18)	0.048 (0.27)		−0.127^**^ (0.06)	−0.236^***^ (0.06)
Business_age^*^log(Size)		−0.614^***^ (0.06)	−0.514^***^ (0.08)		−0.393^***^ (0.04)	−0.341^***^ (0.05)
Business_age^*^SuperHost		0.059 (0.06)	0.264^***^ (0.1)		0.246^***^ (0.04)	0.307^***^ (0.05)
log(Size) ^*^SuperHost		−0.156 (0.19)	0.775^*^ (0.44)		0.149 (0.17)	0.797^***^ (0.28)
Business_age^*^log(Size) ^*^SuperHost			−0.295^**^ (0.13)			−0.205^**^ (0.08)
Year dummies	Yes	Yes	Yes	Yes	Yes	Yes
Wald chi-square (p)	0	0	0			
N	1740	1740	1740	1740	1740	1740

*Standard errors in parentheses*.

* *p<0.10*;

** *p<0.05*;

*** *p<0.01*.

**Figure 1:**
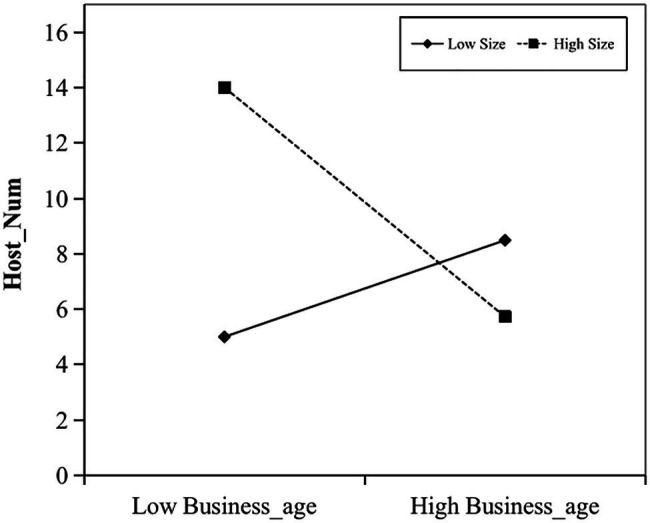
Interaction of business age and size on host performance.

Hypothesis 2 indicates that business age has a greater influence on performance in smaller-sized hosts than larger-size ones. In model 2, the interaction of *Business_age* and *Size* has a significantly negative effects on *Host_Num* (*β*=−0.614, *p*<0.01). Figure 1 shows that the impact of *Business_age* on *Host_Num* increases when *Size* is low, but decreases when *Size* is high. Hypothesis 2 was supported. This result shows that for an inexperienced host, it is beneficial to operate fewer rooms within his/her capacity. As the business age increases, the host has more expertise to improve his/her performance. However, it is more likely to be retrieved by consumers when a host rents out multiple rooms. Thus, junior hosts with several rooms could have better performance. As the business age increases, renting several rooms would make it easier for hosts to attend to one thing and lose sight of another, which is detrimental to their business performance in the long run.

Hypothesis 3 indicates that host reputation can strengthen the influence of business age on host performance. In model 2, the interaction of *Business_age* and *SuperHost* does not has a significant effect on *Host_Num*. Hypothesis 3 was rejected. Therefore, contrary to Hypothesis 3, host reputation does not significantly affect the relationship between business age and host performance.

**Figure 2:**
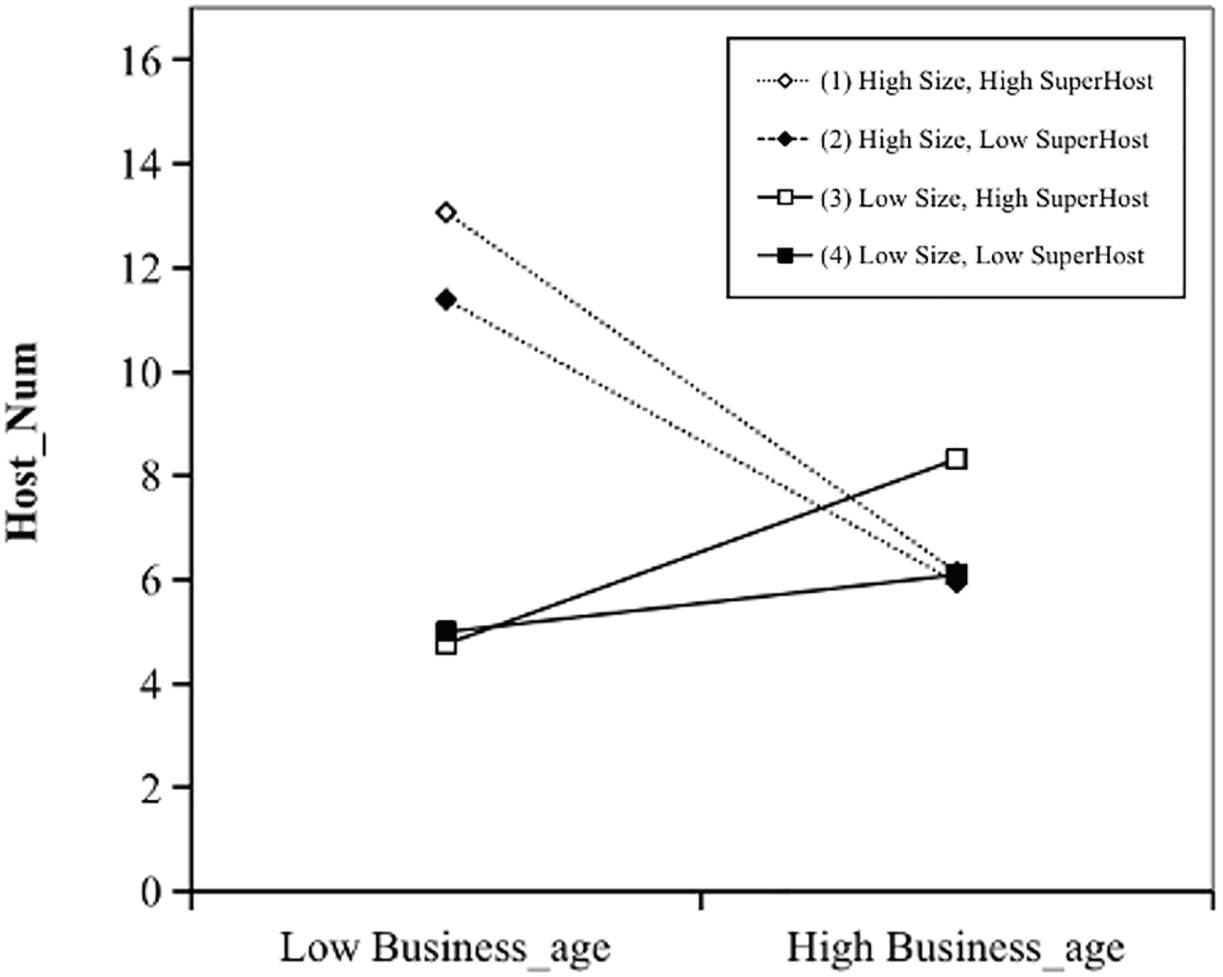
Three-way interaction of business age, size, and host reputation on host performance.

Hypothesis 4 illustrates the three-way interaction effect of business age, size, and host reputation on host performance. Model 3 shows that the three-way interaction in term of *Business_age*, *Size*, and *SuperHost* has a significantly negative coefficient on *Host_Num* (*β*=−0.295, *p*<0.05. The model also suggests that the interaction between *Business_age* and *Size* has a significantly negative coefficient on *Host_Num* (*β*=−0.514, *p*<0.01); the interaction of *Business_age* and *SuperHost* has a significantly positive coefficient on *Host_Num* (*β*=0.264, *p*<0.01); and the interaction of *Size* and *SuperHost* has a significantly positive coefficient on *Host_Num* (*β*=0.775, *p*<0.1). Figure 2 indicates that an increase in *Business_age* on *Host_Num* is significantly enhanced when *Size* is low and *SuperHost* is high, but is significantly decreased when both *Size* and *SuperHost* are high. Hypothesis 4 has been supported. Results toward hypothesis 4 show that the relationship between business age and host performance is strongest among younger hosts possessing higher levels of size and reputation. In addition, the results also show that when size is involved, host reputation plays a positive moderating role in the relationship between business age and host performance. All the empirical results can be seen in Table 3.

Then, we use an alternative estimation method as the robustness check. As shown in Table 3, models 1 to 3 map the main models of negative binomial regressions, while models 4 to 6 show the robustness checks of OLS regressions. In OLS regression models, we take a log transformation on the dependent variable *Host_Num* with skewed distribution (skewness=5.886). As a result, the robustness check models have consistent coefficients with main models.

## Discussion

The tourism and accommodation industries have long been a fertile field for entrepreneurial activities due to their low barriers to entry (Nikraftar and Hosseini, 2016; Li et al., 2020). However, in the field of accommodation sharing, little attention has been paid to entrepreneurial research. Previous studies have shown that shared platforms provide a suitable platform for entrepreneurship (Cohen and Sundararajan, 2015), especially in the field of accommodation. Based on the signaling theory and the resource-based theory, this study investigated the influence of the business age on host performance in the area of accommodation sharing and the three-way interaction effect of business age, size, and host reputation. As shown in Table 4, except for hypothesis 3, our hypotheses 1, 2, and 4 are all supported.

**Table 4:** Test of hypotheses.

	Hypotheses	Results
H1	The host’s business age is positively associated with host performance.	Supported
H2	The relationship between business age and host performance is moderated by size, such that the relationship is stronger for lower size.	Supported
H3	The relationship between business age and host performance is moderated by host reputation, such that the relationship is stronger for higher host reputation.	Not Supported
H4	Business age, size, and host reputation have a three-way interaction effect on host performance, such that the association between business age and host performance will be strongest when size is smaller and host reputation is higher.	Supported

First, in terms of direct effect of business age on host performance, our findings are consistent with Anderson and Eshima (2013) and Karadag (2017). Moreover, it is also cemented by studies from traditional firms (Sørensen and Stuart, 2000; Chang et al., 2002). In the context of accommodation sharing, accumulated knowledge and experiences of the host are vital for hotel operating that can influence host performance. Specifically, the positive impact of business age on host performance suggests that observation, imitation, and learning are crucial for the continuous operation of the host. Accumulated experience and knowledge in customer management, cost control, platform recommendation algorithms, advertising, and taxation can promote host performance. This view is from a learning perspective and has been recognized by Harvie et al. (2010) and Othman and Rosli (2011).

In addition to explaining the direct effects of business age, it has also identified the moderating impact of the resource configuration (business size and host reputation) on host performance from the perspective of resource-based theory. Specifically, business age significantly influences performance of hosts with smaller-sized business than that with larger-sized ones. The results suggest that size smallness is not always a liability for performance, but also an asset for business age. In addition, the impact of business age on host performance increases when size is low, but decreases when size is high, potentially because that the host performance in sharing accommodation depends heavily on human capital. That is, hosts with smaller-sized business are required of lower management and serviceability, making it easier to satisfy consumers, operate business successfully, and enhance performance growth. However, hosts with larger-sized business always manage multiple rooms alone, which can easily increase management difficulties and weaken the service level for every single room, gradually leading to negative influences on performance. It may also be that hosts with larger-sized business have less flexible ability to cope with market changes, and some lists fail to operate, resulting in declining performance. Firm age is closely related to firm size (Coad et al., 2013). Among the extant literature, Kilenthong et al. (2016) pointed out that when taking firm age into consideration, firm size would obviously influence market behavior, that is, firm size can moderate firm age and firm performance. Meanwhile, our research results are also consistent with Bates (2005).

Unexpectedly, it is also discovered that host reputation does not significantly influence the relationship between business age and host performance. According to a study on firm reputation, young firms’ reputation is found to be unstable (Flanagan and O’Shaughnessy, 2005). So, in the early stage of a host’s start-up, high reputation can be easily destroyed by other factors. However, in the later stage of a host’s entrepreneurship, business age and the “superhost” which convey quality signals, could be mutually confirmed, so the individual influence of the host’s reputation may be lower. In addition, the negative moderating effect of size may limit the positive moderating effect of reputation, and reputation effect thereby is not significant.

Finally, the result of the three-way interaction estimates is of vale to gain a deeper understanding on the joint effect of business age, size, and host reputation on customer perception. The joint effect suggests that hosts with smaller-size business have a flexible advantage, giving rise to the performance growth if they pursue business age with a high level of host reputation.

### Theoretical Implications

This study has the following theoretical contributions. First, the business age and performance of companies have long been discussed over the past 50years, yet no consensus has been reached. This study, focusing on accommodation sharing industry, concluded that the business age positively affects host performance in the accommodation sharing. The results show that the accumulation of experience and knowledge is vital for the labor-intensive industry like shared accommodation, which is different from the previous view that young firms are more risk aware, innovative, and flexible, and thus have higher performance (Shane and Venkataraman, 2000; Zahra et al., 2006; Withers et al., 2011). This study therefore can supplement the existing research on firm age and performance.

Second, this study agrees with the view that SMEs are restricted in both tangible and intangible resources (Thornhill and Amit, 2003). However, unlike Newbert's (2007) statement that intangible resources have greater strategic significance for SMEs, the present study suggests that for small firms in shared accommodation industry, the relationship between business age and host performance is more affected by individual moderating effect of tangible resources or joint moderating of tangible and intangible resources. Notably, tangible resources are a necessary condition. In contrast, the boundary effect of intangible resources is weaker than that of tangible resources. This new finding enriches the research on tangible and intangible resources in digital economy. This study thus clarifies the moderation mechanism of the host’s tangible and intangible resources on the relationship between business age and performance.

Third, most of the micro-research on Airbnb focuses on consumer behavior, and relatively less attention has been drawn to hosts (Sigala, 2016). This study re-explains the business behavior of hosts from the perspective of entrepreneurship. This enriches the research on the objects of accommodation sharing and helps to understand entrepreneurship in the informal economy and sharing economy in a narrow sense.

### Practical Implications

This study has some implications for practical guidelines for accommodation sharing entrepreneurs and platform developers. First, it can help hosts to make correct adjustments to achieve optimal performance growth based on their business age, previous size, and reputation when improving performance or making expansion decisions. Moreover, tangible resources (such as size) are essential than intangible resources (such as reputation) for hosts. The hosts could reduce or expand their room numbers to achieve performance optimization. On this basis, hosts can improve the allocation of intangible resources. Second, this study may also help platform managers better understand hosts’ behaviors and solve hosts’ dilemmas. The accumulation of experience and knowledge in related industries and entrepreneurship help hosts to continue their business. In order to help the junior host to become a mature host faster, platform developers could provide some suggestions based on the comprehensive results of the current business age, size, and reputation. Moreover, platform developers may help the hosts renovate their houses and formulate and provide more appropriate courses and promotional products to help the hosts, thereby promoting the platform’s development. In addition, the platform can provide more free or low-cost search engine optimization services for hosts who rent fewer rooms in the early stages of their start-up.

### Limitations and Future Research

This study also has several limitations which is to be solved in future research. First, the sample used in this study is limited to China. If the model is retested in different environments or cultures, the results may be different. In the future, scholars should further test and verify the findings in different contexts and cultural backgrounds. Second, the data used in this study are mainly based on the Airbnb sharing platform, while a variety of accommodation sharing software have emerged in recent years, such as Golightly, Koala, Meituan, Tujia, and Xiaozhu, which deserve further exploration. That is, as these platforms serve different types of individuals in different countries, future research could focus on participants with diverse backgrounds and further refine the business behaviors of different hosts. Third, traditional hotels have gradually begun to operate on third-party digital platforms, which is bound to have a certain impact on accommodation sharing entrepreneurship (Dogru et al., 2020). Thus, whether the conclusions of this study are also applicable to traditional hotels operated by third-party digital platforms remains to be verified. In addition, our study only uses the resource configurations of size and reputation, while many other resources could be considered as boundary condition. Finally, this study mainly focuses on accommodation sharing, but the sharing economy has spawned micro-entrepreneurs in different fields. Further study on the applicability of the findings in other fields can be conducted. Meanwhile, shared accommodation entrepreneurs can be divided into part-time entrepreneurship and full-time entrepreneurship. These two different types of entrepreneurs have different attitudes toward performance, resulting in different business strategies. Therefore, future research should further discuss this issue.

## Conclusion

Hosts on sharing accommodation platforms are generally non-professionals, meaning that the professionalism and reliability of the services provided can be easily questioned by consumers. Therefore, how platform hosts release signals to show more reliability, attract more consumers, and improve performance are of paramount significance for their business success. However, most of the existing studies focus on the psychological and behavioral aspects of consumers participating in sharing accommodation from the perspective of consumers. There is a huge gap in terms of the relationship between hosts’ resources and entrepreneurial performance from the perspective of hosts’ micro-entrepreneurship. Based on the web data crawled from the Airbnb platform, this study firstly explored how the business age of a host affects his or her performance. Secondly, it explores the moderating role of the tangible resources and intangible resources owned by the hosts in this direct effect. Finally, the impact of the three-way interaction on host performance has also been discussed. In particular, this empirical research found that business size and host reputation do have a significant joint moderating effect on the relationship between business age and host performance. This study not only enriches the empirical research on the micro-entrepreneurship of hots in the sharing accommodation field, but also provides practical implications for platform hosts on how to deploy their own resources to achieve performance optimization.

## Data Availability Statement

Publicly available datasets were analyzed in this study. This data can be found at: http://insideairbnb.com/get-the-data.html.

## Author Contributions

XL and ZX contributed to the current research ideas and design of this study. LT wrote the first draft of the manuscript. ZX performed the statistical analysis and contributed to improve the manuscript. XLy edited the revised manuscript and contributed to avoid language errors. All the authors contributed to the article and approved the submitted version.

## Funding

This study was funded by the National Social Science Fund of China, No. 20AZD059.

## Conflict of Interest

The authors declare that the research was conducted in the absence of any commercial or financial relationships that could be construed as a potential conflict of interest.

## Publisher’s Note

All claims expressed in this article are solely those of the authors and do not necessarily represent those of their affiliated organizations, or those of the publisher, the editors and the reviewers. Any product that may be evaluated in this article, or claim that may be made by its manufacturer, is not guaranteed or endorsed by the publisher.

## References

[ref1] Agiomirgianakis G. M. Magoutas A. I. Sfakianakis G. (2012). Determinants of profitability and the decision-making process of firms in the tourism sector: the case of Greece. Int. J. Decis. Sci. Risk Manag. 4, 294–299. doi: 10.1504/ijdsrm.2012.053381

[ref2] Airbnb (2021). About Us (Online). Airbnb. Available at: https://news.airbnb.com/zh/about-us/ (Accessed July 24, 2021).

[ref3] Aissa S. B. Goaied M. (2016). Determinants of Tunisian hotel profitability: The role of managerial efficiency. Tour. Manag. 52, 478–487. doi: 10.1016/j.tourman.2015.07.015

[ref4] Akbar Y. H. Tracogna A. (2018). The sharing economy and the future of the hotel industry: transaction cost theory and platform economics. Int. J. Hosp. Manag. 71, 91–101. doi: 10.1016/j.ijhm.2017.12.004

[ref5] Amit R. Schoemaker P. J. (1993). Strategic assets and organizational rent. Strateg. Manag. J. 14, 33–46. doi: 10.1002/smj.4250140105

[ref6] Anderson B. S. Eshima Y. (2013). The influence of firm age and intangible resources on the relationship between entrepreneurial orientation and firm growth among Japanese SMEs. J. Bus. Ventur. 28, 413–429. doi: 10.1016/j.jbusvent.2011.10.001

[ref7] Audia P. G. Greve H. R. (2006). Less likely to fail: low performance, firm size, and factory expansion in the shipbuilding industry. Manag. Sci. 52, 83–94. doi: 10.1287/mnsc.1050.0446

[ref8] Barney J. (1991). Firm resources and sustained competitive advantage. J. Manage. 17, 99–120. doi: 10.5771/0935-9915-2004-1-53

[ref9] Barney J. B. (2001). Resource-based theories of competitive advantage: A ten-year retrospective on the resource-based view. J. Manage. 27, 643–650. doi: 10.1177/014920630102700602

[ref10] Bates T. (2005). Analysis of young, small firms that have closed: delineating successful from unsuccessful closures. J. Bus. Ventur. 20, 343–358. doi: 10.1016/j.jbusvent.2004.01.003

[ref11] Benítez-Aurioles B. (2018). Why are flexible booking policies priced negatively? Tour. Manag. 67, 312–325. doi: 10.1016/j.tourman.2018.02.008

[ref12] Bensebaa F. (2004). The impact of strategic actions on the reputation building of e-businesses. Int. J. Retail Distrib. Manag. 32, 286–301. doi: 10.1108/09590550410537999

[ref510] Birch David G. W. The Job Generation Process(1979). University of Illinois at Urbana-Champaign’s Academy for Entrepreneurial Leadership Historical Research Reference in Entrepreneurship. Available at: https://ssrn.com/abstract=1510007

[ref13] Boulding W. Kirmani A. (1993). A consumer-side experimental examination of signaling theory: do consumers perceive warranties as signals of quality? J Cons Res 20, 111–123. doi: 10.1086/209337

[ref14] Capasso A. Gallucci C. Rossi M. (2015). Standing the test of time. Does firm performance improve with age? An analysis of the wine industry. Bus. Hist. 57, 1037–1053. doi: 10.1080/00076791.2014.993614

[ref15] Casais B. Fernandes J. Sarmento M. (2020). Tourism innovation through relationship marketing and value co-creation: A study on peer-to-peer online platforms for sharing accommodation. J. Hosp. Tour. Manag. 42, 51–57. doi: 10.1016/j.jhtm.2019.11.010

[ref16] Cetin G. Bilgihan A. (2016). Components of cultural tourists’ experiences in destinations. Curr. Issues Tour. 19, 137–154. doi: 10.1080/13683500.2014.994595

[ref17] Chang Y. Gomes J. F. Schorfheide F. (2002). Learning-by-doing as a propagation mechanism. Environ. Manage. 92, 1498–1520. doi: 10.1257/000282802762024601

[ref18] Choi Y. R. Ha S. Kim Y. (2021). Innovation ambidexterity, resource configuration and firm growth: is smallness a liability or an asset? Small Bus. Econ. 26, 1–27. doi: 10.1007/s11187-021-00507-3

[ref19] Chu W. Chu W. (1994). Signaling quality by selling through a reputable retailer: An example of renting the reputation of another agent. Marketing Sci. 13, 177–189. doi: 10.1287/mksc.13.2.177

[ref20] Coad A. Rao R. (2010). Firm growth and R&D expenditure. Econ. Innovation New. Tech. 19, 127–145. doi: 10.1080/10438590802472531

[ref21] Coad A. Segarra A. Teruel M. (2013). Like milk or wine: does firm performance improve with age? Struct. Change Econ. Dynam. 24, 173–189. doi: 10.1016/j.strueco.2012.07.002

[ref22] Cohen M. Sundararajan A. (2015). Self-regulation and innovation in the peer-to-peer sharing economy. U. Chi. L. Rev. Dialogue 82:116. doi: 10.31235/osf.io/rqdhw

[ref23] Covin J. G. Slevin D. P. (1991). A conceptual model of entrepreneurship as firm behavior. Entrep. Theory Pract. 16, 7–26. doi: 10.1177/104225879101600102

[ref24] Davidson J. O. C. (2010). New slavery, old binaries: human trafficking and the borders of ‘freedom’. Global Netw. 10, 244–261. doi: 10.1111/j.1471-0374.2010.00284.x

[ref25] Day G. S. Wensley R. (1988). Assessing advantage: a framework for diagnosing competitive superiority. J. Marketing 52, 1–20. doi: 10.1177/002224298805200201

[ref26] Dillahunt T. R. Malone A. R. (2015). “The promise of the sharing economy among disadvantaged communities,” in *Proceedings of the 33rd Annual ACM Conference on Human Factors in Computing Systems*. April 18–23, 2015; Seoul Republic of Korea, 2285–2294.

[ref27] Ditta-Apichai M. Kattiyapornpong U. Gretzel U. (2020). Platform-mediated tourism micro-entrepreneurship: implications for community-based tourism in Thailand. J. Hosp. Tour. Technol. 11, 223–240. doi: 10.1108/jhtt-05-2019-0079

[ref28] Dogru T. Hanks L. Mody M. Suess C. Sirakaya-Turk E. (2020). The effects of Airbnb on hotel performance: evidence from cities beyond the United States. Tour. Manag. 79:104090. doi: 10.1016/j.tourman.2020.104090

[ref29] Dowling G. R. (2016). Winning the Reputation Game: Creating Stakeholder Value and Competitive Advantage. Cambridge, United States: MIT Press.

[ref30] Drover W. Wood M. S. Corbett A. C. (2018). Toward a cognitive view of signalling theory: individual attention and signal set interpretation. J. Manag. Stud. 55, 209–231. doi: 10.1111/joms.12282

[ref31] Ert E. Fleischer A. Magen N. (2016). Trust and reputation in the sharing economy: The role of personal photos in Airbnb. Tour. Manag. 55, 62–73. doi: 10.1016/j.tourman.2016.01.013

[ref32] Ferreira B. S. Morais D. B. Lorscheider M. (2015). Using web marketplaces to reach untapped markets. North Carolina Cooperative Extension Services.

[ref33] Flanagan D. J. O’shaughnessy K. C. (2005). The effect of layoffs on firm reputation. J. Manage. 31, 445–463. doi: 10.1177/0149206304272186

[ref34] Fradkin A. Grewal E. Holtz D. Pearson M. (2015). “Bias and Reciprocity in Online Reviews: Evidence From Field Experiments on Airbnb,” in *16th ACM Conference on Economics and Computation*. July 24-28, 2016; Maastricht, Netherlands.

[ref35] Geroski P. Gugler K. (2004). Corporate growth convergence in Europe. Oxford Econ. Pap. 56, 597–620. doi: 10.1093/oep/gpf055

[ref36] Gibbs C. Guttentag D. Gretzel U. Yao L. Morton J. (2018). Use of dynamic pricing strategies by Airbnb hosts. Int. J. Contemp. Hosp. Manag. 30, 2–20. doi: 10.1108/IJCHM-09-2016-0540

[ref37] Grant R. M. (1991). The resource-based theory of competitive advantage: implications for strategy formulation. Calif. Manag. Rev. 33, 114–135. doi: 10.2307/41166664

[ref38] Grant R. M. (2002). Contemporary Strategy Analysis: Concept, Techniques, Aplicattions. Mssachusetts: Blackwell.

[ref39] Greenwood R. Li S. X. Prakash R. Deephouse D. L. (2005). Reputation, diversification, and organizational explanations of performance in professional service firms. Organ. Sci. 16, 661–673. doi: 10.1287/orsc.1050.0159

[ref40] Hall R. (1992). The strategic analysis of intangible resources. Strateg. Manag. J. 13, 135–144. doi: 10.1002/smj.4250130205

[ref41] Hall E. H. Lee J. (2014). Assessing the impact of firm reputation on performance: an international point of view. Int. Bus. Res. 7, 1–13. doi: 10.5539/ibr.v7n12p1

[ref42] Hamari J. Sjöklint M. Ukkonen A. (2016). The sharing economy: why people participate in collaborative consumption. J. Assoc. Inf. Sci. Technol. 67, 2047–2059. doi: 10.2139/ssrn.2271971

[ref43] Hannan M. T. Freeman J. (1984). Structural inertia and organizational change. Am. Sociol. Rev. 1, 149–164. doi: 10.2307/j.ctvjz813k.7

[ref44] Harvie C. Narjoko D. Oum S. (2010). Firm characteristic determinants of SME participation in production networks. ERIA [Preprint]. Available at: https://core.ac.uk/download/pdf/9306170.pdf

[ref45] Hollick M. Braun P. (2005). “Lifestyle entrepreneurship: the unusual nature of the tourism entrepreneur,” in *Proceedings of the Second Annual AGSE International Entrepreneurship Research Exchange*, February 10–12, 2005; Swinburne Press, Melbourne, 10–11.

[ref46] Huang Q. Chen X. Ou C. X. Davison R. M. Hua Z. (2017). Understanding buyers' loyalty to a C2C platform: the roles of social capital, satisfaction and perceived effectiveness of e-commerce institutional mechanisms. Inf. Syst. J. 27, 91–119. doi: 10.1111/isj.12079

[ref47] Ismail N. A. Jenatabadi H. S. (2014). The influence of firm age on the relationships of airline performance, economic situation and internal operation. Transp. Res. Part A Policy Pract. 67, 212–224. doi: 10.1016/j.tra.2014.06.010, PMID: 32288368 PMC7126140

[ref48] Jhaver S. Karpfen Y. Antin J. (2018). “Algorithmic anxiety and coping strategies of Airbnb hosts,” in *Proceedings of the 2018 CHI Conference on Human Factors in Computing Systems*. April 21-26, 2018; Montreal QC, Canada, 1–12.

[ref49] Kamasak R. (2017). The contribution of tangible and intangible resources, and capabilities to a firm’s profitability and market performance. Eur. J. Manag. Bus. Econ. 26, 252–275. doi: 10.1108/EJMBE-07-2017-015

[ref50] Karadag H. (2017). The impact of industry, firm age and education level on financial management performance in small and medium-sized enterprises (SMEs): evidence from Turkey. J. Entrepreneurship Emerg. Econ. 9, 300–314. doi: 10.1108/jeee-09-2016-0037

[ref51] Kilenthong P. Hultman C. M. Hills G. E. (2016). Entrepreneurial marketing behaviours: impact of firm age, firm size and firm’s founder. J. Res. Mark. Entrepreneurship 18, 127–145. doi: 10.1108/jrme-05-2015-0029

[ref52] Kim W. G. Li J. J. Brymer R. A. (2016). The impact of social media reviews on restaurant performance: The moderating role of excellence certificate. Int. J. Hosp. Manag. 55, 41–51. doi: 10.1016/j.ijhm.2016.03.001

[ref53] Koh E. King B. (2017). Accommodating the sharing revolution: a qualitative evaluation of the impact of Airbnb on Singapore’s budget hotels. Tour. Recreat. Res. 42, 409–421. doi: 10.1080/02508281.2017.1314413

[ref54] Kor Y. Y. Mesko A. (2013). Dynamic managerial capabilities: configuration and orchestration of top executives’ capabilities and the firm’s dominant logic. Strateg. Manag. J. 34, 233–244. doi: 10.1002/smj.2000

[ref55] Kristiansen S. Furuholt B. Wahid F. (2003). Internet cafe entrepreneurs: pioneers in information dissemination in Indonesia. Int. J. Entrepreneurship Innov. 4, 251–263. doi: 10.5367/000000003129574315

[ref56] Lam L. W. Chuang A. Wong C.-S. Zhu J. N. (2019). A typology of three-way interaction models: applications and suggestions for Asian management research. Asia Pacific J. Manag. 36, 1–16. doi: 10.1007/s10490-018-9577-9

[ref57] Lee D. (2016). How Airbnb short-term rentals exacerbate Los Angeles's affordable housing crisis: analysis and policy recommendations. Harvard Law Policy Rev. 10, 229–255.

[ref58] Li Y. Huang S. S. Song L. (2020). Opportunity and necessity entrepreneurship in the hospitality sector: examining the institutional environment influences. Tour. Manag. Perspect. 34:100665. doi: 10.1016/j.tmp.2020.100665

[ref59] Li J. Moreno A. Zhang D. J. (2019). “Agent pricing in the sharing economy: Evidence from Airbnb,” in Sharing Economy. Springer Series in Supply Chain Management. Vol 6. ed. M. Hu (Cham: Springer), 485–503.

[ref60] Liang S. Schuckert M. Law R. (2017). Multilevel analysis of the relationship between type of travel, online ratings, and management response: empirical evidence from international upscale hotels. J. Travel Tour. Mark. 34, 239–256. doi: 10.1080/10548408.2016.1156613

[ref61] Liang S. Schuckert M. Law R. Chen C.-C. (2020). The importance of marketer-generated content to peer-to-peer property rental platforms: evidence from Airbnb. Int. J. Hosp. Manag. 84:102329. doi: 10.1016/j.ijhm.2019.102329

[ref62] Lussier R. N. (1995). A nonfinancial business success versus failure prediction model for young firms. J. Small Bus. Manag. 33, 8–20.

[ref63] Lutz C. Newlands G. (2018). Consumer segmentation within the sharing economy: The case of Airbnb. J. Bus. Res. 88, 187–196. doi: 10.1016/j.jbusres.2018.03.019

[ref64] Magno F. Cassia F. Ugolini M. M. (2018). Accommodation prices on Airbnb: effects of host experience and market demand. TQM J. 30, 608–620. doi: 10.1108/TQM-12-2017-0164

[ref66] Martin C. J. (2016). The sharing economy: A pathway to sustainability or a nightmarish form of neoliberal capitalism? Ecol. Econ. 121, 149–159. doi: 10.1016/j.ecolecon.2015.11.027

[ref67] Mason C. H. Perreault W. D. Jr. (1991). Collinearity, power, and interpretation of multiple regression analysis. J. Marketing Res. 28, 268–280. doi: 10.1177/002224379102800302

[ref68] Molloy J. C. Barney J. B. (2015). Who captures the value created with human capital? A market-based view. Acad. Manag. Perspect. 29, 309–325. doi: 10.5465/amp.2014.0152

[ref69] Nahapiet J. Ghoshal S. (1998). Social capital, intellectual capital, and the organizational advantage. Acad. Manag. Rev. 23, 242–266. doi: 10.2307/259373

[ref70] Newbert S. L. (2007). Empirical research on the resource-based view of the firm: an assessment and suggestions for future research. Strateg. Manag. J. 28, 121–146. doi: 10.1002/smj.573

[ref71] Nieto García M. Muñoz-Gallego P. A. Viglia G. Gonzalez-Benito O. (2020). Be social! The impact of self-presentation on peer-to-peer accommodation revenue. J. Travel Res. 59, 1268–1281. doi: 10.1177/0047287519878520

[ref72] Nikraftar T. Hosseini E. (2016). Factors affecting entrepreneurial opportunities recognition in tourism small and medium sized enterprises. Tour. Rev. 71, 6–17. doi: 10.1108/TR-09-2015-0042

[ref73] Nunes P. M. Gonçalves M. Serrasqueiro Z. (2013). The influence of age on SMEs’ growth determinants: empirical evidence. Small Bus. Econ. 40, 249–272. doi: 10.1007/s11187-011-9363-2

[ref74] Oskam J. Boswijk A. (2016). Airbnb: the future of networked hospitality businesses. J. Tour. Futures 2, 22–42. doi: 10.1108/JTF-11-2015-0048

[ref75] Othman P. Rosli M. M. (2011). The impact of tourism on small business performance: empirical evidence from Malaysian islands. Int. J. Bus. Soc. Sci. 2, 11–21.

[ref76] Parasuraman A. Zeithaml V. A. Berry L. (1988). SERVQUAL: A multiple-item scale for measuring consumer perceptions of service quality. J. Retail. 64, 12–40.

[ref77] Phizacklea A. Ram M. (1995). Ethnic entrepreneurship in comparative perspective. Int. J. Entrepreneurial Behav. Res. 1, 48–54. doi: 10.1108/13552559510079760

[ref78] Priporas C.-V. Stylos N. Rahimi R. Vedanthachari L. N. (2017). Unraveling the diverse nature of service quality in a sharing economy: A social exchange theory perspective of Airbnb accommodation. Int. J. Contemp. Hosp. Manag. 29, 2279–2301. doi: 10.1108/IJCHM-08-2016-0420

[ref79] Rajan R. G. Zingales L. (1995). What do we know about capital structure? Some evidence from international data. J. Finance 50, 1421–1460. doi: 10.1111/j.1540-6261.1995.tb05184.x

[ref80] Ranger-Moore J. (1997). Bigger may be better, but is older wiser? Organizational age and size in the New York life insurance industry. Am. Sociol. Rev. 62, 903–920. doi: 10.2307/2657346

[ref81] Resnick P. Zeckhauser R. Swanson J. Lockwood K. (2006). The value of reputation on eBay: A controlled experiment. Exper Econ. 9, 79–101. doi: 10.1007/s10683-006-4309-2

[ref82] Richardson P. S. Dick A. S. Jain A. K. (1994). Extrinsic and intrinsic cue effects on perceptions of store brand quality. J. Marketing 58, 28–36. doi: 10.1177/002224299405800403

[ref83] Rogers M. (2004). Networks, firm size and innovation. Small Bus. Econ. 22, 141–153. doi: 10.1023/B:SBEJ.0000014451.99047.69

[ref84] Serrasqueiro Z. S. Nunes P. M. (2008). Performance and size: empirical evidence from Portuguese SMEs. Small Bus. Econ. 31, 195–217. doi: 10.1007/s11187-007-9092-8

[ref85] Shane S. Venkataraman S. (2000). The promise of entrepreneurship as a field of research. Acad. Manag. Rev. 25, 217–226. doi: 10.1007/978-3-540-48543-8_8

[ref86] Sigala M. (2016). Learning with the market: A market approach and framework for developing social entrepreneurship in tourism and hospitality. Int. J. Contemp. Hosp. Manag. 28, 1245–1286. doi: 10.1108/IJCHM-06-2014-0285

[ref87] Slevin D. P. Covin J. G. (1997). Strategy formation patterns, performance, and the significance of context. J. Manage. 23, 189–209. doi: 10.1177/014920639702300205

[ref88] Sørensen J. B. Stuart T. E. (2000). Aging, obsolescence, and organizational innovation. Adm. Sci. Q. 45, 81–112. doi: 10.2307/2666980

[ref89] Spence M. (1978). “Job market signaling,” in Uncertainty in Economics. eds. P. Diamond and M. Rothschild (Netherland: Elsevier), 281–306.

[ref90] Srivastava R. K. Fahey L. Christensen H. K. (2001). The resource-based view and marketing: The role of market-based assets in gaining competitive advantage. J. Manage. 27, 777–802. doi: 10.1177/014920630102700610

[ref91] Stamboulis Y. Skayannis P. (2003). Innovation strategies and technology for experience-based tourism. Tour. Manag. 24, 35–43. doi: 10.1016/S0261-5177(02)00047-X

[ref92] Steffens P. Davidsson P. Fitzsimmons J. (2009). Performance configurations over time: implications for growth–and profit–oriented strategies. Entrep. Theory Pract. 33, 125–148. doi: 10.1111/j.1540-6520.2008.00283.x

[ref93] Stinchcombe A. (1965). Organization-creating organizations. Society 2, 34–35.

[ref94] Tayles M. Pike R. H. Sofian S. (2007). Intellectual capital, management accounting practices and corporate performance: perceptions of managers. Account. Audit. Account. J. 20, 522–548. doi: 10.1108/09513570710762575

[ref95] Teubner T. Hawlitschek F. Dann D. (2017). Price determinants on Airbnb: how reputation pays off in the sharing economy. J. Self-Governance Manag. Econ. 5, 53–80. doi: 10.22381/jsme5420173

[ref96] Thornhill S. Amit R. (2003). Learning about failure: bankruptcy, firm age, and the resource-based view. Organ. Sci. 14, 497–509. doi: 10.1287/orsc.14.5.497.16761

[ref97] Van Praag C. M. (2003). Business survival and success of young small business owners. Small Bus. Econ. 21, 1–17. doi: 10.1023/A:1024453200297

[ref98] Venkataraman S. Van De Ven A. H. Buckeye J. Hudson R. (1990). Starting up in a turbulent environment: A process model of failure among firms with high customer dependence. J. Bus. Ventur. 5, 277–295. doi: 10.1016/0883-9026(90)90006-F

[ref99] Viglia G. Minazzi R. Buhalis D. (2016). The influence of e-word-of-mouth on hotel occupancy rate. Int. J. Contemp. Hosp. Manag. 28, 2035–2051. doi: 10.1108/IJCHM-05-2015-0238

[ref100] Wang D. Nicolau J. L. (2017). Price determinants of sharing economy based accommodation rental: A study of listings from 33 cities on Airbnb. Com. Int. J. Hosp. Manag. 62, 120–131. doi: 10.1016/j.ijhm.2016.12.007

[ref101] Wei J. Ouyang Z. Chen H. (2017). Well known or well liked? The effects of corporate reputation on firm value at the onset of a corporate crisis. Strateg. Manag. J. 38, 2103–2120. doi: 10.1002/smj.2639

[ref102] Wells J. D. Valacich J. S. Hess T. J. (2011). What signal are you sending? How website quality influences perceptions of product quality and purchase intentions. MIS Q. 35, 373–396. doi: 10.2307/23044048

[ref103] Wernerfelt B. (1984). A resource-based view of the firm. Strateg. Manag. J. 5, 171–180. doi: 10.1002/smj.4250050207

[ref104] Wiklund J. Baker T. Shepherd D. (2010). The age-effect of financial indicators as buffers against the liability of newness. J. Bus. Ventur. 25, 423–437. doi: 10.1016/j.jbusvent.2008.10.011

[ref105] Wiklund J. Shepherd D. (2003). Knowledge-based resources, entrepreneurial orientation, and the performance of small and medium-sized businesses. Strateg. Manag. J. 24, 1307–1314. doi: 10.1002/smj.360

[ref106] Williamson O. E. (1975). Markets and hierarchies: analysis and antitrust implications: a study in the economics of internal organization. *University of Illinois at Urbana-Champaign’s Academy for Entrepreneurial Leadership Historical Research Reference in Entrepreneurship*.

[ref107] Withers M. C. Drnevich P. L. Marino L. (2011). Doing more with less: the disordinal implications of firm age for leveraging capabilities for innovation activity. J. Small Bus. Manag. 49, 515–536. doi: 10.1111/j.1540-627X.2011.00334.x

[ref108] Wu P. C. Wang Y. C. (2011). The influences of electronic word-of-mouth message appeal and message source credibility on brand attitude. Asia Pac. J. Mark. Logist. 23, 448–472. doi: 10.1108/13555851111165020

[ref109] Xie K. Heo C. Y. Mao Z. E. (2021). Do professional hosts matter? Evidence from multi-listing and full-time hosts in Airbnb. J. Hosp. Tour. Manag. 47, 413–421. doi: 10.1016/j.jhtm.2021.04.016

[ref110] Yacouel N. Fleischer A. (2012). The role of cybermediaries in reputation building and price premiums in the online hotel market. J. Travel Res. 51, 219–226. doi: 10.1177/0047287511400611

[ref111] Yao L. Li J. Li J. (2020). Urban innovation and intercity patent collaboration: A network analysis of China’s national innovation system. Technol. Forecast. Soc. Change 160:120185. doi: 10.1016/j.techfore.2020.120185

[ref112] Zahra S. A. Sapienza H. J. Davidsson P. (2006). Entrepreneurship and dynamic capabilities: A review, model and research agenda. J. Manag. Stud. 43, 917–955. doi: 10.1111/j.1467-6486.2006.00616.x

